# Biomechanical Effect of UHMWPE and CFR-PEEK Insert on Tibial Component in Unicompartmental Knee Replacement in Different Varus and Valgus Alignments

**DOI:** 10.3390/ma12203345

**Published:** 2019-10-14

**Authors:** Yong-Gon Koh, Hyoung-Taek Hong, Kyoung-Tak Kang

**Affiliations:** 1 Joint Reconstruction Center, Department of Orthopaedic Surgery, Yonsei Sarang Hospital, 10 Hyoryeong-ro, Seocho-gu, Seoul 06698, Korea; osygkoh@gmail.com; 2 Department of Mechanical Engineering, Yonsei University, 50 Yonsei-ro, Seodaemun-gu, Seoul 03722, Korea; hyoungtaekhong@gmail.com

**Keywords:** computational model, biomechanics, unicompartmental knee replacement, UHMWPE, CFR-PEEK, varus and valgus alignments

## Abstract

The current study aims to analyze the biomechanical effects of ultra-high molecular weight polyethylene (UHMWPE) and carbon-fiber-reinforced polyetheretherketone (CFR-PEEK) inserts, in varus/valgus alignment, for a tibial component, from 9° varus to 9° valgus, in unicompartmental knee replacement (UKR). The effects on bone stress, collateral ligament force, and contact stress on other compartments were evaluated under gait cycle conditions, by using a validated finite element model. In the UHMWPE model, the von Mises’ stress on the cortical bone region significantly increased as the tibial tray was in valgus >6°, which might increase the risk of residual pain, and when in valgus >3° for CFR-PEEK. The contact stress on other UHMWPE compartments decreased in valgus and increased in varus, as compared to the neutral position. In CFR-PEEK, it increased in valgus and decreased in varus. The forces on medial collateral ligaments increased in valgus, when compared to the neutral position in UHMWPE and CFR-PEEK. The results indicate that UKR with UHMWPE showed positive biomechanical outputs under neutral and 3° varus conditions. UKR with CFR-PEEK showed positive biomechanical outputs for up to 6° varus alignments. The valgus alignment should be avoided.

## 1. Introduction

Unicompartmental knee replacement (UKR) has become a popular alternative to total knee replacement (TKR), owing to favorable patient satisfaction reports and functional outcomes [1,2]. With a more functional anatomy being maintained, UKR can provide faster recovery and better restoration of knee kinetics, in comparison to TKR [3]. The long-term survival rates of UKR have been considerably improved, owing to refined surgical techniques and strict patient selection [4].

Various studies have shown that inaccurate alignment or technical errors in prosthetic components might cause early wear of polyethylene, periprosthetic fractures, and high revision rates to correct residual pain that is caused by implant failure [5,6,7]. The main complications are caused by abnormal tibial bone stress, which is thought to result from coronal malalignments of the tibial component. Therefore, UKR can be considered as a more technically demanding surgical treatment. In addition, numerous authors have suggested the relative malalignment of the knee in medial UKR, to avoid progressive osteoarthritis (OA) in the opposite compartment [8,9,10]. The effects of knee alignment on the long-term outcome of UKR have received attention in some reports [10,11].

The wear of ultra-high molecular weight polyethylene (UHMWPE), in both fixed- and mobile-bearings, continues to be a significant factor in the long-term performance of UKR [12]. Alternative polymers to replace UHMWPE in UKR or TKR have been proposed and they include the use of carbon-fiber reinforced polyetheretherketone (CFR-PEEK). In most cases, the carbon fibers in the CFR-PEEK materials are small, chopped fibers, and generally have no specific orientation in the polyetheretherketone (PEEK) matrix. CFR-PEEK has been classified as a low wear material in a recent in-vitro experimental study, being suitable for high-conformity scenarios, such as total hip replacement and conforming UKR [13]. However, it was recently stated that the wear rates of CFR-PEEK were very high and almost two orders of magnitude higher than the wear rate of ultra-high molecular weight polyethylene (UHMWPE) under comparable conditions for total knee replacement (TKR) [14]. S. C. Scholes and A. Unsworth stated that UKR with CFR-PEEK exhibited lower volumetric wear rates than those that were previously found in conventional metal-on-UHMWPE prostheses when tested under similar conditions [15]. Further study is required to confirm CFR-PEEK as an alternative material to UHMWPE. However, to the best of our knowledge, there has been a lack of research on the tibia bone stress when using CFR-PEEK tibial insert materials, which is an important factor determining aseptic loosening and anteromedial pain.

Most frequently, UKR is performed at a perpendicular coronal tibial position. However, various studies have reported improved results upon placing the tibial component parallel to the line of natural tibial joint, or within a few varus degrees of the epiphyseal axis [16,17]. There has been no study on the biomechanical effects of CFR-PEEK when compared to UHMWPE, with respect to varus and valgus alignments in the tibial component. Moreover, as mentioned above, there has been no biomechanical justification for the UHMWPE insert in the varus and valgus alignments in the tibial component.

The purpose of this study is to evaluate the biomechanical effects of the various varus/valgus alignment positions of tibial components in UKR with different UHMWPE and CFR-PEEK insert materials. We evaluate the contact stress on other compartments, the bone stress, and collateral ligament force, under a gait cycle condition, by using models from 9° varus to 9° valgus, with an increment of 1°, which is centered on the neutral position.

## 2. Materials and Methods

### 2.1. Intact Knee Joint Model

The present study was conducted while using a fully validated finite element (FE) model of knee joint [18,19,20,21]. The intact knee was made from computed tomography (CT) and magnetic resonance imaging (MRI) of the left knee joint of a healthy 35-year-old male volunteer. The CT imaging was conducted with a slice thickness of 0.1 mm, while using a 64-channel CT scanner (Somatom Sensation 64, Siemens Healthcare, Erlangen, Germany). In addition, MRI imaging was conducted using a 3.0 T MRI scanner (Achieva 3.0T; Philips Healthcare, North Brabant, The Netherlands) and a custom-designed knee joint cardiac coil [21]. MRI scans with a slice thickness of 0.4 mm were obtained in the sagittal plane. A high-resolution environment was used for the spectral pre-saturation inversion recovery (SPIR) sequence (TE, 25.0 ms; TR, 3590.8 ms; acquisition matrix, 512 pixels × 512 pixels; NEX, 2.0; field-of view, 140 mm × 140 mm) and a 3-T MRI system (Discovery MR750w^®^, GE Healthcare, Milwaukee, WI, USA) [21].

**Figure 1:**
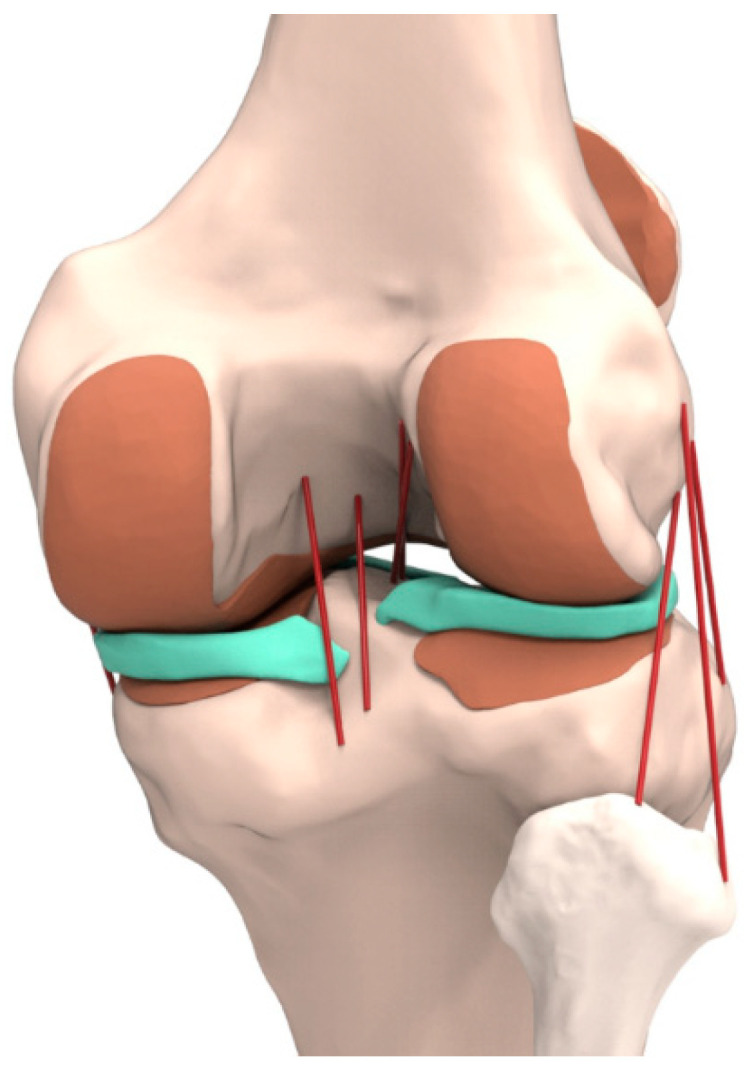
Developed three-dimensional finite element (3D FE) knee joint model from medical image.

The process of combining the reconstructed CT and MRI models through the positional alignment of each model was conducted while using the commercial software Rapid form (version 2006; 3D Systems Korea, Seoul, Korea). Subsequently, the image data were imported into the image-processing software Mimics 17.0 (Materialise Ltd., Leuven, Belgium) to extract the geometry used for the generation of the three-dimensional (3D) models of all structures. The initial graphics exchange specification (IGES) files that were exported from Mimics were entered into Unigraphics NX (version 7.0; Siemens PLM Software, Torrance, CA, USA) to form solid models for each femur, tibia, fibula, patella, and soft-tissue segment (Figure 1). The solid model was imported into Hypermesh (version 8.0; Altair Engineering, Troy, MI, USA) to generate an FE mesh. Afterwards, the FE mesh was analyzed while using the ABAQUS software (version 6.11; Simulia, Providence, RI, USA).

In the model, the bones were assumed rigid, whereas the tibia was considered transversely isotropic as it was stiffer than soft tissue and had a minimal influence in the present study [22]. Constitutive laws were assumed for the tibia cortical and cancellous bones [23,24]. The cortical bone was considered to be transversely isotropic. The third axis was considered as parallel to the anatomical axis of each bone. The cancellous bone was considered as linear isotropic [23,24]. The articular cartilage was assumed to be an isotropic, linear elastic material, owing to the time-independent and simple compressive load applied to the knee joint [25]. The menisci were assumed to be a transversely isotropic, linearly elastic, homogeneous material [26]. The applied material properties were as shown in Table 1.

To represent the meniscal attachments, each meniscal horn was bonded to the bone using linear spring elements (SPRINGA element type) with a total stiffness of 2000 N/mm [27]. In addition, the major ligaments were modeled while using nonlinear and tension-only spring elements [28,29]. The force-displacement relationship, based on the functional bundles in the actual ligament anatomy, is expressed in the following:

$$f\left(\varepsilon \right)=\{\begin{array}{c} \frac{k\varepsilon^{2}}{4\varepsilon_{1}},\;0\leq \varepsilon \leq 2\varepsilon_{1}\\ k\left(\varepsilon -\varepsilon_{1}\right),\;\varepsilon >2\varepsilon_{1}\\ 0,\;\varepsilon <0 \end{array}$$

$$\varepsilon =\frac{l-l_{0}}{l_{0}}$$

$$l_{0}=\frac{l_{r}}{\varepsilon_{r}+1}$$

where f(ε) is the current force, *k* the stiffness, ε the strain, and *ε*_1_ is assumed to be a constant equal to 0.03. The ligament bundle slack of length *l*_0_ was calculated while using the reference bundle length *l_r_* and the reference strain *ε_r_* in the upright reference position.

The interfaces between the cartilage and bones were modeled as being fully bonded. The contacts between the femoral cartilage and meniscus, meniscus and tibial cartilage, and femoral cartilage and tibial cartilage were modeled for both the medial and lateral sides, and resulted in six contact pairs [18]. The contacts at all articulations adopted a finite sliding, frictionless hard contact algorithm with no penetration [18]. Convergence was defined as a relative change of more than 5% between the two adjacent meshes [20].

### 2.2. UKR Model

A fixed-bearing UKR (Zimmer, Inc., Warsaw, IN, USA) was virtually implanted in the medial compartment of the developed normal knee model. The bone models were imported and appropriately positioned, trimmed, and meshed with rigid elements by using surgical techniques [30].

**Figure 2:**
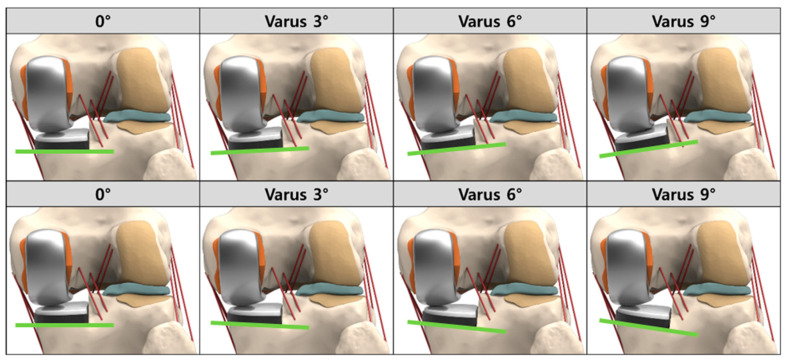
Schematic of varus/valgus conditions for tibial insert.

Based on the dimensions of the femur and tibia, size 6 and 5 devices were chosen for the femoral and tibial components, respectively. The devices were aligned with the mechanical axis and positioned at the medial edge of the tibia. The neutrally aligned tibial baseplate was defined as having a square (0°) inclination in the coronal plane with a 5° posterior slope. The rotating axis was defined as a line parallel to the lateral edge of the tibial component passing through the center of the femoral component peg. A neutral femoral component distal cut, which was perpendicular to the mechanical axis of the femur and parallel to the tibial cut, was reproduced. The 19 different cut surfaces of the medial tibial plateau were created with different inclinations in the coronal plane (from 9° varus to 9° valgus, in increments of 1°) (Figure 2).

With respect to the implanted model, a cement gap of 1 mm was simulated between the bone and component. The femoral and tibial components, bone cement, and tibia insert (UHMWPE and CFR-PEEK) were modeled as linear elastic isotropic materials [31,32,33,34,35]. The materials of the femoral component, tibia insert, tibial component, and bone cement corresponded to a cobalt chromium alloy (CoCr), UHMWPE, CFR-PEEK, a titanium alloy (Ti6Al4V), and poly(methyl methacrylate) (PMMA), respectively. Compared to pure PEEK, CFR-PEEK contains a 30% proportion of carbon fiber reinforcement [35].

In terms of Young’s modulus and Poisson’s ratio, the material properties were shown in Table 2 [31,32,33,34,35]. The friction coefficients between the articulating surfaces were assumed to be 0.07 for UHMWPE and 0.04 for CFR-PEEK in accordance with the range that was reported in the literature [33,36,37].

### 2.3. Loading and Boundary Conditions

The FE investigation included two types of loading conditions, which corresponded to the loads that were used in the experimental part of the study, for the validation of the UKR model and its predictions under gait cycle loading conditions.

A validation of the intact model was conducted in a previous study, and the UKR model was validated through a comparison with models in previous experimental studies [18,19,20,21,38]. The validation of the UKR model was conducted for the flexion angles of 0°, 30°, 60°, and 90°, by using a passive flexion simulation. Additionally, anterior and posterior drawer loads of 134 N were separately applied to the tibia at the knee center, in a manner that is similar to that adopted in a previous experimental study [38].

**Figure 3:**
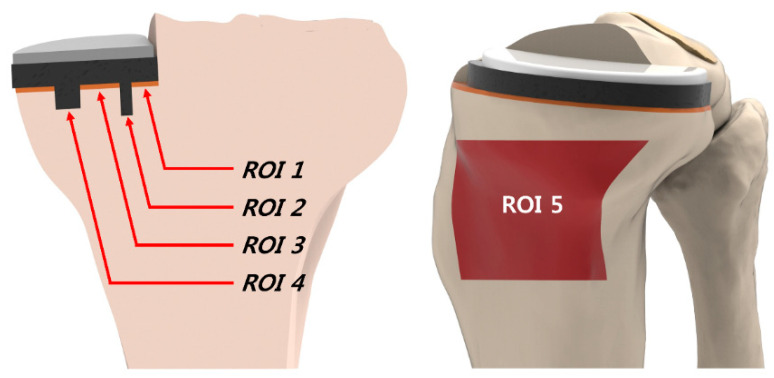
Five locations for the interests of this study.

Gait cycle loading was applied as a second loading to compare the biomechanical effects of the three different tibial insert materials [39]. A computational analysis was conducted while using the force controls of the anterior-posterior force with respect to the compressive load applied to the hip [40,41]. A proportional-integral derivative controller was incorporated into the computational model to make allowance for the control of the quadriceps, in a manner that is similar to that in an existing experiment [42]. Internal-external and varus/valgus torques were applied to the tibia [40,41]. We analyzed the contact stress on the lateral meniscus and tibial cartilage in the other compartments, as well as the tibial bone stress, which influences residual pain. Additionally, we analyzed the collateral ligament force. We defined five regions of interest (ROIs) on the proximal tibia (Figure 3). ROIs 1 and 4 were defined at the resection corner between the sagittal and transverse tibia bone cuts. The other three ROIs were located on the cancellous bone surface, beneath the tibial baseplate, with ROIs 2 and 4 being lateral and medial to the keel slot, respectively. ROI 5 was defined to investigate the source of residual pain. For all models, ROI 5 was located on the proximal anteromedial cortical bone surface.

## 3. Results

### 3.1. FE Model Validation

**Figure 4:**
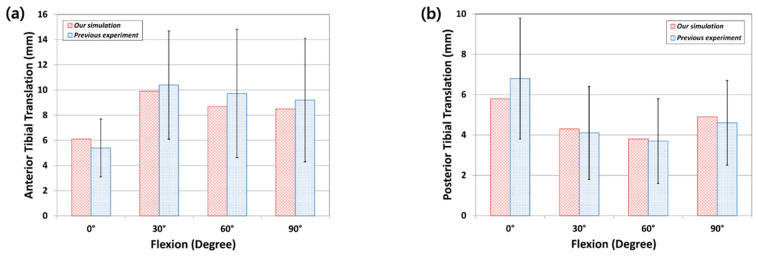
Comparison with previous experimental study for validation of unicompartmental knee replacement (UKR) model: (**a**) anterior tibial translation; and, (**b**) posterior tibial translation.

In a previous study, an intact model was validated and explained while using an in vivo laxity test for a subject identical to the FE model. We compared it with the results of the experiment on our own subject to validate the intact FE model. The anterior tibial translation was calculated at 2.83 mm in the experiment, and at 2.54 mm in the FE model under the 30° flexion loading condition; the posterior tibial translation was calculated at 2.12 mm in the experiment and 2.18 mm in the FE model for validation. Good agreement was observed between the experimental results and the FE model [19]. The UKR model validation was conducted while using the anterior and posterior tibial translations in the anterior and posterior drawer tests at 134 N for 6.1, 9.9, 8.7, and 8.5 mm, and 5.8, 4.3, 3.8, and 4.9 mm at 0°, 30°, 60°, and 90° knee flexion, respectively (Figure 4). These show good agreement with previous experimental data within the value ranges under the anterior and posterior drawer loadings [38].

### 3.2. Comparison of Contact Stress on Other Compartments in UHMWPE and CFR-PEEK Tibial Component in Varus/Valgus Alignment

**Figure 5:**
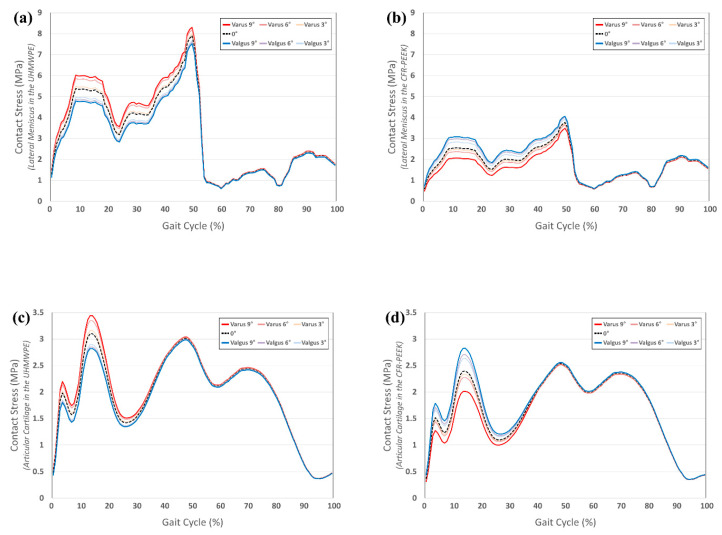
Comparison of contact stress on other compartments in varus/valgus condition with respect to different tibial insert materials during gait cycle: (**a**) lateral meniscus in the ultra-high molecular weight polyethylene (UHMWPE); (**b**) lateral meniscus in the carbon-fiber reinforced polyetheretherketone (CFR-PEEK); (**c**) articular cartilage in the UHMWPE; and, (**d**) articular cartilage in the CFR-PEEK.

Figure 5 shows the contact stress on the other compartments (articular cartilage and lateral meniscus) in the UHMWPE and CFR-PEEK tibial insert with the varus/valgus tibial component. The contact stress on the articular cartilage and lateral meniscus increased in varus and decreased in valgus, in comparison to the neutral position in the UHMWPE insert. However, the contact stress on the articular cartilage and lateral meniscus decreased in varus and increased in valgus, in comparison to the neutral position of the CFR-PEEK insert. There was different contact stress during the stance phase in the UHMWPE and CFR-PEEK insert models. For the UHMWPE model, it increased by 11% and 8% for varus 9°, and decreased by 11% and 9% for valgus 9° in the lateral meniscus and articular cartilage, respectively, in comparison to the neutral position. For the CFR-PEEK model, it increased by 21% and 18% for valgus 9°, and decreased by 19% and 16% for varus 9° in the lateral meniscus and articular cartilage, respectively, in comparison to the neutral position.

### 3.3. Comparison of Von-Mises Stress in Tibial Bone and Collateral Ligament Force in UHMWPE and CFR-PEEK Tibial Component in Varus/Valgus Alignment

**Figure 6:**
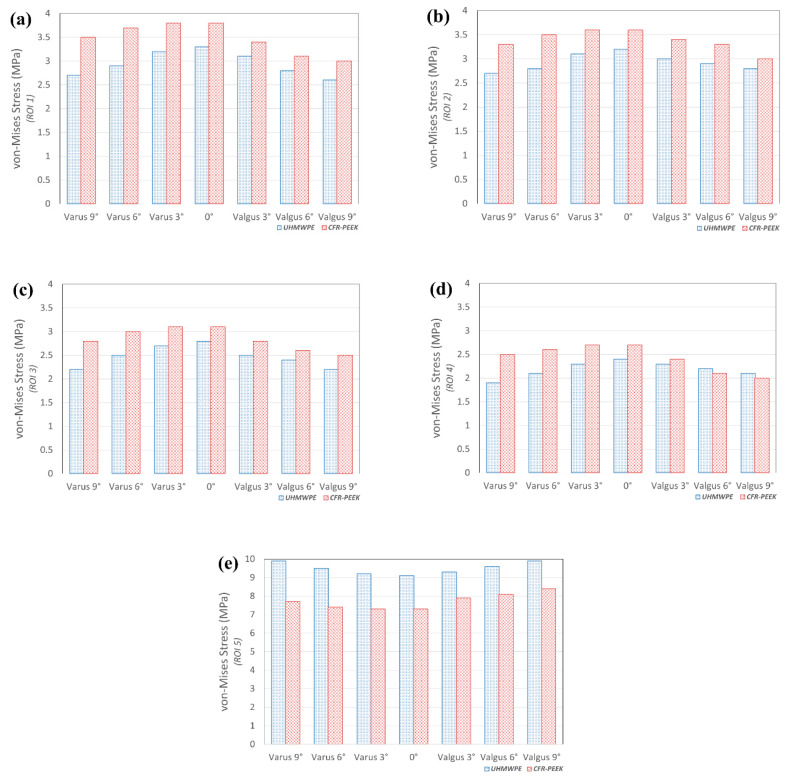
Comparison of von-Mises stress in ROI 1–5 in varus/valgus condition with respect to different tibial insert materials during gait cycle: (**a**) ROI 1; (**b**) ROI 2; (**c**) ROI 3; (**d**) ROI 4; and, (**e**) ROI 5.

**Figure 7:**
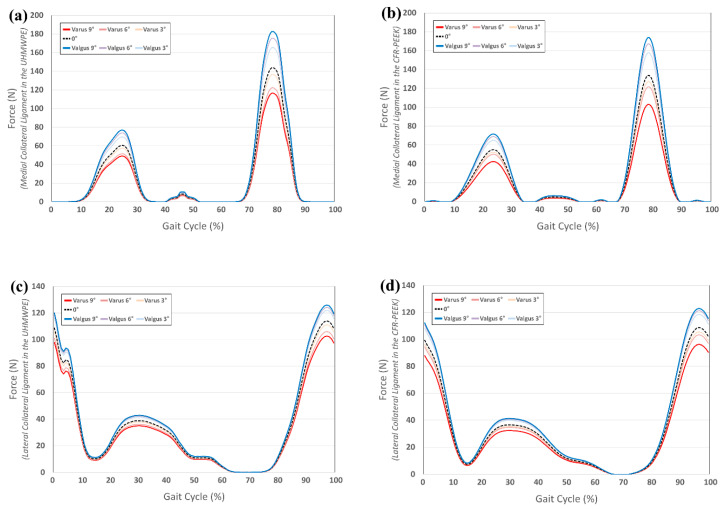
Comparison of ligament force in varus/valgus condition with respect to different tibial insert materials during gait cycle: (**a**) medial-collateral ligament in the UHMWPE; (**b**) medial-collateral ligament in the CFR-PEEK; (**c**) lateral-collateral ligament in the UHMWPE; and, (**d**) lateral-collateral ligament in the CFR-PEEK.

Figure 6 indicates the von-Mises stress on the tibia bone in the UHMWPE and CFR-PEEK tibial inserts with the varus/valgus tibial component. The greatest stress was exerted on ROI 1 and 2 (cancellous bone), in both the UHMWPE and CFR-PEEK. This trend did not change with varus and valgus in the tibial component. In addition, the bone stress in ROI 1–4, in the CFR-PEEK, was higher than in the UHMWPE. The von-Mises stress on the UHMWPE in the tibial bone showed a similar value from the neutral position to the varus 3° model. The CFR-PEEK model showed a similar value from the neutral position to varus 6°; however, it rapidly decreased afterwards, under the valgus condition. The stress in ROI 5 (cortical bone) with UHMWPE was greater than with CFR-PEEK. In addition, a similar trend was observed in ROI 1–4, for both UHMWPE and CFR-PEEK, under the varus and valgus conditions. Figure 7 indicates the force on the collateral ligament in the UHMWPE and CFR PEEK tibial insert with a varus/valgus tibial component. The force on the medial collateral ligament with the UHMWPE and CFR-PEEK increased under the valgus condition, in comparison to the neutral position. The force in the UHMWPE model with valgus 9° increased by 19% in the medial- collateral ligament, as compared to the neutral position. Moreover, the force on the medial-collateral ligament increased more in the CFR-PEEK model under the valgus condition. The force in the UHMWPE model with valgus 9° increased by 27% in the medial collateral ligament, in comparison to the neutral position. A complex pattern was observed in the lateral collateral ligament for the UHMWPE and CFR-PEEK. Under the valgus conditions, the force on the lateral collateral ligament increased during the swing and stance phases, respectively.

## 4. Discussion

The positive biomechanical effect that was observed in the UHMWPE tibial insert at the neutral position and with slight varus malalignment was the most important finding of the current study. Additionally, for the CFR-PEEK tibial insert, there was no problem with a varus malalignment greater than that of the UHMWPE tibial insert; however, the valgus malalignment should be avoided.

The precise restoration of the mechanical axis and correct implant positioning are major contributors to the improvement of implant longevity and the clinical outcomes of UKR. There is still no general agreement in terms of the optimal position of the tibial component [43].

This study aimed to evaluate the optimal tibial alignment for UKR with UHMWPE and CFR-PEEK tibial inserts, and the potential aberrant performances due to the malalignment of the tibial components. An FE model was used to analyze and compare different combinations of several biomechanical outputs. In addition, the FE model was impractical in the experimental evaluation of progressive OA in the other compartments and tibia bone stress problems, with respect to different insert materials under the varus/valgus conditions. Furthermore, the advantage of computational simulations that use a single subject is that the effects of tibial component alignment and the differences in insert materials, within the same subject, can be determined, except for variables, such as weight, height, bone geometry, differences in ethnicity and sex, ligament properties, and component size [44]. The intact knee model underwent a series of validation steps. The results showed good agreement with previous experimental data, in terms of kinematics and contact area, as demonstrated by the FE analysis for the same subject. In addition, an intact and a UKR model were both validated by using experimental and kinematics data. Therefore, the UKR model developed in the current study, along with the following analysis, can be considered to be reasonable.

As mentioned above, the UHMWPE insert led to result variation in the coronal positioning of the tibial tray. Sawatari et al. [45] analyzed the orientation influence of the unicompartmental tibial component on cancellous bone stresses in the coronal and sagittal planes, and correlated these findings to clinical data. They suggested that a slight valgus inclination of a UKR tibial component is better than a varus or square inclination in the coronal plane. In addition, Isekawa et al. [46] analyzed the inclination influence of the UKR tibial component on stress, in the proximal tibia, and the influence of contact pressure on the metal–bone interface, in the identical femorotibial alignment. They suggested that a slight valgus inclination of the tibial component might be better than varus or even a 0° (square) inclination, as the stress distribution is related. Therefore, a slight valgus inclination is preferable for both. Simpson et al. [47] found that the inclination angles had a minimal effect on the bone strain in the Oxford UKR, with the exclusion of the 2° varus inclination. However, there is a limitation in the three models that are mentioned above, as they only model tibia and not femur or other bony structure and soft tissues such as cartilage and ligament. Recently, Zhu et al. [48] developed a UKR model with entirely bony structure and soft tissue. They investigated the coronal inclination angles of the tibial tray ranging from 10° varus to 10° valgus. They suggested a range from 4° varus to 4° valgus for the inclination of the tibial component in the mobile-bearing UKR. In addition, Innocenti et al. [49] evaluated the biomechanical effects of different varus/valgus alignment tibial component positions in UKR. They proposed that a neutral mechanical or 3 varus alignment exhibits similar biomechanical outputs in the bone, collateral ligament strain, and polyethylene insert. A 6° varus alignment or changes in the valgus alignment were always associated with more detrimental effects. The two studies mentioned above also showed limitations, as they only evaluated the biomechanical effect under static conditions. However, UKR is a surgical treatment for younger patients; therefore, it requires analysis under dynamic loading conditions, such as gait cycles.

We studied the contact stress on other compartments, the stress on the tibial bone and the force on the collateral ligament under a gait cycle. Our results showed that alignment influences the development of OA in other compartments. Previous studies that have attempted to evaluate the influence of alignment were limited due to their short follow-up and absence of accurate knee alignment measurements [10,11,50]. In addition, a previous clinical study only used a radiography parameter (joint space narrowing) for the development of OA in the opposite compartment. This narrowing of joint space shows the articular cartilage loss in the joint and it is considered to be a more reliable marker of OA than the osteophytes or subchondral sclerosis. However, the radiography parameter might cause results to vary, owing to scanning inaccuracies and human error. We evaluated contact stress to investigate the progressive OA in other compartments because the contact parameters are known to be closely related to the degenerative OA of the knee joint [18,51]. An intriguing finding was that the effect of tibial malalignment was different for the different tibial insert materials. In UHMWPE, the contact stress on other compartments decreased in valgus and slightly increased under varus conditions [52]. This trend proved to be in fine agreement with a previous study. The contact force on the other compartment decreased under the valgus condition using UHMWPE and in the cadaveric experiment [52]. However, the CFR-PEEK model it increased in valgus and decreased in varus. Additionally, the difference between contact stresses on other compartments, in both the UHMWPE and CFR-PEEK model, was found during the stance phase. This proved that the contact stress was mainly influenced by the axial force.

The cortical bone always exerted the majority of the load transferred by the tibial baseplate, as its elastic modulus is much larger than that of the cancellous bone. An abnormally high stress of the proximal medial cortical bone was used to understand the cause of persistent pain after UKR [47]. Additionally, the aseptic loosening of prosthetic components, and especially that of the tibial component, is one of the major failure modes in UKR [53]. Excessive stress in both the cortical and cancellous bone cause it, and it leads to stress shielding. The major reason of the latter is the significant difference in the Young’s modulus between the bone and tibial baseplate material. In contrast to TKR, the interface between the tibial baseplate and the tibia is significantly smaller. This leads to bone stresses being more sensitive to malalignment and malpositioning of component.

This UHMWPE model showed higher stress in the cortical bone region and lower stress in the cancellous bone region, in comparison to the CFR-PEEK model. The tibial insert material was only changed to CFR-PEEK; however, the stress-shielding effect was reduced because the material properties of CFR-PEEK were similar to the bone’s material properties. The increase of bone stresses in the cortical bone and the decrease of bone stresses in the cancellous bone around the tibial baseplate may cause pain, which is induced by a loosening of the tibial baseplate [54,55,56,57]. Stress in the cortical bone increased, while, in the cancellous bone, it decreased under the valgus condition for both UHMWPE and CFR-PEEK, in comparison to the neutral position. In other words, the valgus malalignment should be avoided in both UHMWPE and CFR-PEEK.

After medial UKR was implanted, the valgus deformity was induced, even though the neutral knee model had a normal alignment (valgus rotation 0°) [49,58]. This phenomenon can be explained by the different stiffness between the medial and lateral compartments of the UKR knee. On the lateral side, the cartilage of both the femur and tibia had an elastic modulus of 15 MPa. In contrast to the medial side elastic moduli of the tibial insert materials UHMWPE and CFR-PEEK, the elastic modulus was much higher than that of the cartilage. Consequently, the Young’s Modulus of the medial and lateral compartments differed by more than one order of magnitude; therefore, the deformation of materials in each compartment depended on their elastic modulus [54]. In a previous study, the valgus deformity that was induced by the strain loading, in the medial-collateral ligament, increased, while the strain in the lateral collateral ligament decreased [54]. Hence, whilst a surgeon balances a knee during UKR in an unloaded state, the knee will no longer be balanced once it is loaded [54].

Our study confirmed this result. The force on the medial-collateral ligament also increased for the UHMWPE and CFR-PEEK models under the valgus condition. This trend was particularly observed in CFR-PEEK. The reason was that CFR-PEEK has a higher elastic modulus than UHMWPE, as mentioned previously. Therefore, the difference in material properties became greater here than in other areas. Our results showed that progressive OA could occur in other compartments under the valgus condition. Additionally, valgus should be avoided because the MCL force rapidly increases in the CFR-PEEK model.

There are several strengths to this study. First, the FE model included the femur, tibia, and related soft tissues in the present study [31,45,46,47]. Second, gait cycle loading, and complex vertical static loading condition was applied in this study [31,45,46,47,48,49,54]. Third, this study validated not just the initial FE model, but also achieved kinematic validation of the UKR FE models with experimental data [31,45,46,47,48,49,54].

Nevertheless, there are three limitations. First, the FE model represented a fixed bearing UKR, and it cannot apply other implant designs, such as the mobile bearing UKR. Second, a single coefficient of friction value was used, when there is in fact a variety in the coefficients of friction for different materials. The effect of different values will be investigated in future studies.

Third, patient satisfaction and clinical results could not be assessed by the results from FE analysis. However, contact stress on the other compartments, force exerted on ligaments, and bone stresses are important factors that should be investigated for the evaluation of biomechanical effects in computational biomechanics [18,19,20,21,31,45,46,47,48,49].

## 5. Conclusions

The biomechanical justifications for the tibial insert materials UHMWPE and CFR PEEK were evaluated to confirm the suitability of various tibial component alignment positions while using an FE model. We performed a simulation for other compartments to investigate progressive OA. Contact stress on other compartments increased in varus and decreased in valgus for UHMWPE. In CFR-PEEK, an opposite trend was observed, by which the stress-shielding effect was less than that of UHMWPE, because its elastic modulus was closer to that of bone. However, in the CFR-PEEK model, a significant negative biomechanical effect was observed, owing to the valgus condition, in comparison to UHMWPE. In this model, the stress on the cortical and cancellous bone increased and decreased rapidly under the valgus and varus condition, respectively, in comparison to the neutral position. This trend was also observed in UHMWPE; however, the negative biomechanical effects were more pronounced in CFR PEEK; here, the varus allowance was wider than in UHMWPE, as there was less difference in the biomechanical effects from the neutral position to varus 6°. Such a trend could be found in the medial collateral ligament. The force on the medial collateral ligament became greater under the valgus condition of the CFR-PEEK model, in comparison to the UHMWPE model. The UHMPWE model, with a neutral position or minor varus (3°) alignment, exhibits similar biomechanical outputs in terms of the contact stress on other compartments, the stress on the bone, and the collateral ligament force. Therefore, the neutral position and minor varus conditions are recommended in the UHMWPE model. In addition, a greater varus condition of up to 6° is recommended in the CFR-PEEK model, as it showed similar biomechanical output. However, the valgus condition should be avoided in the CFR-PEEK model.

## Figures and Tables

**Table 1:** Material properties for the 3D FE knee joint model.

Material	Material Properties
Cortical bone [23,24]	E_1_ = E_2_ = 11.5 GPa E_3_ = 17 GPa ν_12_ = 0.51, ν_23_ = ν_13_ = 0.31
Cancellous bone [23,24]	E = 2.13 GPa ν = 0.3
Articular cartilage [25]	E = 15 MPa ν = 0.47
Menisci [26]	E_c_ = 120 MPa (in the circumferential direction) E_a_ = E_r_ = 20 MPa MPa (in the axial and radial directions.) v_cr_ = 0.3 (for axial directions) v_ar_ = v_ac_ = 0.3 (for circumferential and radial directions)

**Table 2:** Material properties for the implanted model.

Materials	Material Properties
CoCr	E = 195 GPa v = 0.3
Ti6Al4V	E = 110 GPa v = 0.3
UHMWPE	E = 685 MPa v = 0.47
CFR-PEEK	E = 18000 MPa v = 0.4
PMMA	E = 1940 MPa and v = 0.4
